# Effect of Prebiotic Polysaccharides on the Rheological Properties of Reduced Sugar Potato Starch Based Desserts

**DOI:** 10.3390/polym12102224

**Published:** 2020-09-27

**Authors:** Dorota Gałkowska, Monika Południak, Mariusz Witczak, Lesław Juszczak

**Affiliations:** 1 Department of Food Analysis and Evaluation of Food Quality, Faculty of Food Technology, University of Agriculture in Krakow, 30-149 Krakow, Poland; dorota.galkowska@urk.edu.pl (D.G.); mjpoludniak@gmail.com (M.P.); 2 Department of Engineering and Machinery for Food Industry, Faculty of Food Technology, University of Agriculture in Krakow, 30-149 Krakow, Poland; rrwitcza@cyf-kr.edu.pl; 3 Department of Dietetics and Food Science, Faculty of Science & Technology, Jan Dlugosz University in Czestochowa, 42-200 Czestochowa, Poland

**Keywords:** low sugar dessert, starch, inulin, polydextrose, rheological properties

## Abstract

The aim of the study was to assess the possibility of using polysaccharides: inulin and polydextrose in combination with steviol glycosides as sucrose substitutes in starch-based desserts with reduced sugar content and to determine their influence on the rheological properties of these desserts. The samples (starch-milk desserts) were prepared from native potato starch, milk, dye, flavouring agent, and sucrose. The sucrose was partially or completely substituted with steviol glycosides and inulin or polydextrose. The rheological evaluation of the desserts was performed by determining pasting characteristics, viscosity curves, creep and recovery curves and mechanical spectra. Substitution of sucrose with prebiotic polysaccharides modified the rheological characteristics of the starch-milk desserts to a degree depending on the type and level of the substituting agent. Inulin reduced the peak viscosity of starch-milk paste, while it had no effect on the final viscosity of the product, contrary to polydextrose, which increased value of the latter parameter. The desserts exhibited a non-Newtonian, shear-thinning flow behaviour. The use of inulin, in both the highest and the lowest concentrations, significantly changed the consistency coefficient and the flow index values, while such a phenomenon was not observed in the case of polydextrose. The desserts with inulin showed increased values of the storage modulus and reduced susceptibility to stress, manifesting strengthened viscoelastic structure. The results indicate that the both prebiotic polysaccharides can serve as substitutes for sucrose in desserts with reduced sugar contents.

## 1. Introduction

Starch, a natural biopolymer, is a reserve plant material. It is also a renewable raw material, commonly used in the food industry and other industrial branches [1]. Starch is insoluble in cold water, but when it is heated in water, it thickens to form a colloidal solution called starch paste. It is characterized by a high viscosity, and in the case of its sufficient high concentration it forms a gel after cooling [2]. The rheological characteristics of starch pastes and gels are mainly a function of a temperature and time of hydrothermal processing [3]. One of the most important functional properties of starch is its thickening capacity. For this reason, it is an excellent ingredient for making a desired consistency of a product, which also significantly affects its sensory characteristics [4]. In addition to natural starches, chemically modified ones that constitute food additives are widely used in food processing. They show better functional properties than natural starches [1,5,6,7]. Presence of chemically modified starches in the product may, however, cause consumer distrust of the product. Therefore, other solutions for creating the consistency of products are highly demanded. A possible alternative to modifying the rheological characteristics of products by chemically modified starches seems to be the use of starch-non-starch polysaccharide interactions [8,9,10].

Natural or modified starches are commonly used thickeners and texture creating agents in milk-based desserts [11,12,13]. The ingredient that determines the taste of such desserts is sucrose, which is the most popular sweetener used in food processing. It gives the products sweet taste, and at the same time it possesses structure-forming, binding, filling and preserving properties.

It is well known that excessive consumption of sucrose promotes the development of diseases, such as obesity, diabetes and tooth decay. For this reason, many consumers are interested in low-sugar or sugar-free foods, including desserts [13]. In the production of such foods, sucrose is replaced with low-energy sweeteners of high sweetening capacity. Unfortunately, many of these substances are synthetic compounds, not always accepted by consumers. Therefore, it is desirable to use natural, low-energy sweeteners. Such substances include steviol glycosides (E 960) isolated from the leaves of *Stevia rebaudiana* Bert plant [14]. Their consumption does not increase blood glucose levels and insulin sensitivity. In addition, they can be used in the diet of people with phenylketonuria, because they are not a source of phenylalanine. They do not increase energetic value of food products or dishes, and at the same time, they do not significantly modify their taste. Additionally, due to their thermal stability, they can be used in the production of food subjected to heat treatment [14,15].

In addition to the use of high-intensity sweeteners, reduction of the sucrose content in many food products also requires the use of bulking agents. One of the most important factors influencing consumer acceptance of a food product is its texture, which can be created or controlled with the use of food ingredients or additives. Such substances are, among others, inulin and polydextrose. Due to their texturing properties, low energy value and prebiotic properties, they are desirable food ingredients [16]. Inulin is a carbohydrate plant reserve material, belonging to the long-chain fructans composed of fructose monomers linked by β-(2→1)-glycosidic bonds. It is not digested in the digestive tract, but it is partially or fully fermented by *Lactobacillus* spp. and *Bifidobacterium* spp. in the colon to produce short chain fatty acids [17]. The low pH value of the colon content contributes to a restriction of putrefactive processes and protection against colitis and cancer [17]. In food technology, inulin is used as a filling, emulsifying, thickening, stabilizing, or replacing sucrose and fat agent [18]. Polydextrose (E 1200), in turn, is a synthetic, branched polymer which is produced by polycondensation of glucose. It consists of randomly connected low- and high-molecular weight glucose polymers that contain different types of glycosidic bonds. Polydextrose shows good water solubility and stability during processing [19]. This polymer is not digested by the body’s endogenous enzymes and it has prebiotic properties. It is partially metabolized by the microbiota of the large intestine to form short-chain fatty acids which are absorbed later on [19]. Polydextrose is used as sugar substitute in low-calorie or diabetic food products, in which it serves as a humectant, stabiliser, filling or thickening agent.

Since substituting of sucrose with small amount of intense sweetener results in modification of rheological characteristics of low- or reduced-sugar products, the aim of this study was to assess the effect of partial or complete replacement of sucrose with prebiotic polysaccharides, namely inulin or polydextrose, on the rheological characteristics of starch-milk desserts. These polysaccharides were selected because both of them exhibit prebiotic properties, but differ in terms of their chemical moiety as well as structure which may have a significant impact on the rheological characteristics of the desserts.

## 2. Materials and Methods

### 2.1. Materials

The samples were starch-milk desserts being prepared from potato starch (Superior Standard; Wielkopolskie Przedsiębiorstwo Przemysłu Ziemniaczanego S.A., Luboń, Poland) and from other ingredients, namely powdered defatted agglomerated milk (Dairy Cooperative in Gostyn, Gostyń, Poland), sucrose (Diamant; Pfeifer & Langen S.A., Gostyń, Poland), steviol glycosides (Pure Reb-A; Stevija, the Netherlands), curcumin (E 100; JAR—Jaskulski Aromaty, Warszawa, Poland), vanilla flavouring agent (Delecta—Rieber Foods S.A., Włocławek, Poland), inulin GR (DP ≥ 10; Beneo Orafti, Tienen, Belgium), polydextrose (E 1200; Litesse; Danisco, Copenhagen, Denmark).

The desserts were prepared according to the recipes given in Table 1, as follows. Milk powder was reconstituted according to the producer’s instruction (250 mL of water per 20 g of milk powder). Starch, sucrose, flavouring agent (five drops) and curcumin (three drops) were added to the milk and the mixture was intensively mixed. Twenty-five grams of the suspension was transferred to an aluminum pan of a Rapid Visco Analyzer (RVA; TecMaster, Perten Instruments, Hägersten, Sweden) and subjected to the pasting procedure as described in Section 2.2.1. The resulting dessert was considered as a control. The other samples were prepared by substituting 1/3 or 2/3 or 3/3 of sucrose by weight with appropriate amounts of steviol glycosides (their sweetness was set as 300:1 as compared to sucrose), and inulin (IN33 or IN67 or IN100, respectively) or polydextrose (PD33 or PD76 or PD100, respectively).

### 2.2. Methods

#### 2.2.1. Pasting Characteristics Measurement

Pasting properties were determined using a Rapid Visco Analyzer (RVA; TecMaster, Perten Instruments, Hägersten, Sweden). Milk suspensions of starch with all the other ingredients (as described in Section 2.1) were subjected to heating-cooling treatment at constant stirring of 160 rpm, as follows: heating from 50 to 95 °C at the rate of 12 °C/min, holding at 95 °C for 2.5 min, cooling to 50 °C at the rate of 12 °C/min, and holding at 50 °C for 2 min. The following RVA parameters were determined: PT—pasting temperature (°C); PV—peak viscosity (mPa·s); HPV—hot paste viscosity (mPa·s); BD—viscosity breakdown (BD = PV − HPV; mPa·s); FV—final viscosity (mPa·s); SB—viscosity setback (SB = FV − HPV; mPa·s). The resulting starch pastes were immediately proceeded to further rheological measurements.

#### 2.2.2. Rheological Measurement

The rheological characterisation of the starch deserts was performed using a Mars II rheometer (Thermo Fisher Scientific Inc., Karlsruhe, Germany) with cone-plate measuring geometry (diameter 35 mm, angle 2°, gap size 0.105 mm). Viscosity curves were determined at 25 °C at increasing shear rate from 1 1/s to 100 1/s, and described by a power law model (1):

(1) $$\eta_{ap}=K\cdot {\dot{\gamma }}^{n-1}$$

where: *η_ap_*—apparent viscosity (Pa·s), $\dot{\gamma }$—shear rate (1/s), *K*—consistency coefficient (Pa·s^n^), *n*—flow index (dimensionless).

The creep and recovery tests were performed at 25 °C. The constant shear stress in the creep phase amounted to 1.0 Pa in the range of stress to strain proportionality. The creep and recovery phases lasted for 150 and 300 s, respectively. The resulting curves of compliance as a function of time were described by the Burgers model of four parameters (2 and 3), which consists of the Maxwell and the Kelvin models placed in series:

(2) $$J\left(t\right)=J_{0}+J_{1}\left[1-exp\left(\frac{-t}{\lambda_{ret}}\right)\right]+\frac{t}{\eta_{0}},\;\mathrm{for}\;t\;\leq \;t1$$

(3) $$J\left(t\right)=\frac{t_{1}}{\eta_{0}}-J_{1}\left[1-exp\left(\frac{-t_{1}}{\lambda_{ret}}\right)\right]\cdot \;exp\left(\frac{-t}{\lambda_{ret}}\right),\;\mathrm{for}\;t\;>\;t1$$

where: *J(t)*—compliance (1/Pa), *J*_0_—instantaneous compliance (1/Pa), *J*_1_—viscoelastic compliance (1/Pa), *λ_ret_*—retardation time (s), *η*_0_—zero shear viscosity (Pa·s), *t*—real time of experiment (s), *t*_1_—time when the constant stress is removed (s).

A sweep frequency test was performed in the linear viscoelastic range at the constant stain value of 0.03 and in the angular frequency range of 1–100 rad/s. The mechanical spectra were plotted in terms of elastic modulus (*G*′) and viscous modulus (*G*″) as a function of angular frequency (*ω*). The following power law equations were fitted to the experimental data:

(4) $$G{}^{\prime }=K{}^{\prime }\cdot \omega^{n{}^{\prime }}\;$$

(5) $$G{}^{\prime \prime }=K{}^{\prime \prime }\cdot \omega^{n{}^{\prime \prime }}\;$$

where: *G*′—storage modulus (Pa), *G*″—loss modulus (Pa), *ω*—angular frequency (rad/s), *K*′, *K*″—experimental constants, *n*′, *n*″—slopes of the plots.

#### 2.2.3. Statistical Analysis

All the experiments were performed in triplicate. The results are presented as mean ± standard deviation. Two-way analysis of variation (ANOVA) followed by the Fisher’s test to establish the least significant difference (LSD) were performed using Statistica 9.0 software (StatSoft Inc., Tulsa, OK, USA). Significance level was set at 0.05 and statistical differences were considered as significant for *p* < 0.05. The Pearson linear correlation coefficients for selected pairs of rheological parameters were also determined.

## 3. Results and Discussion

### 3.1. Pasting Characteristics

**Figure 1:**
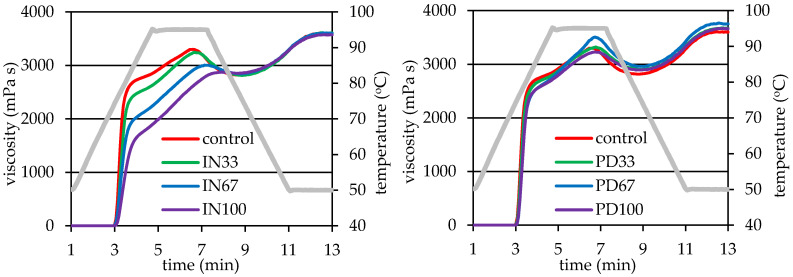
Pasting curves of potato starch in systems forming starch-milk desserts: without steviol glycosides (control), with inulin (IN33, IN67, IN100), with polydextrose (PD33, PD67, PD100).

Viscoelastic structure of milk-starch dessert is formed as a result of heating while stirring and then cooling the milk suspension of starch and other dessert ingredients. Substantial factors determining the structure of the final product and its characteristics perceived by consumers are mainly the amount and kind of starch, interactions between starch and other food components, and thermal processing conditions [13]. Pasting curves of the analyzed systems forming starch-milk desserts are shown in Figure 1, and the pasting parameters determined from these curves are given in Table 2. The pasting of starch in the presence of non-starch ingredients of the desserts involved a sharp increase in viscosity after exceeding the pasting temperature and reaching viscosity maximum at 95 °C. Then a slight decrease in viscosity and again its increase during cooling period was observed. The characteristic phenomenon that distinguishes the pasting curves of the tested systems from a typical pasting curve of starch suspended in water is an intensive increase in viscosity of the system within the initial period of heating, followed by a gentle increase in viscosity until its maximum is reached [13]. The maximum viscosity of starch paste reflects the ability of starch granules to freely swell before their physical disintegration [2]. Taking into account relatively high concentration of the starch in the analyzed samples, it might be concluded that the observed maximum viscosity values are lower than those ones reported for potato starch [2].

This indicates that a restriction in swelling of the starch granules occurred in the systems due to the higher starch concentration (and thus, less water available for the starch granules) and from the presence of non-starch components, i.e., sucrose and/or inulin or polydextrose, as well as milk. As a result, the tested systems achieved the maximum viscosity not before a temperature of 95 °C, which is much higher than that achieved by potato starch pasting in water [2]. Interactions between milk proteins and starch and between sucrose and starch significantly affect rheological properties of the final product [13,20]. The effect of the above-mentioned interactions is governed by many factors, such as protein concentration, thermal processing conditions and presence of other compounds in the systems [21,22]. As a result of study on the influence of milk proteins on the rheological characteristics of starch, Matignon et al. [22] found that changes in the achieved viscosity values may be related to the modification of its swelling capacity and/or surface properties resulting from the presence of protein in the system. According to Kumar et al. [23], casein proteins and whey proteins differently affect the rheological properties of starch systems, i.e., casein molecules capable of forming larger aggregates tend to be physically adsorbed on the surface of starch grains, while whey proteins are mainly located in the dispersing phase.

Substitution of part of sucrose amount with prebiotic polysaccharides had different effect on the starch pasting (Table 2). The pasting temperature values remained at similar level as in the control sample and, at the same time, no significant statistical variation in the values of this parameter was found. The above results are in contradiction to those given by Wang et al. [10], where the presence of inulin increased the starch pasting temperature. In turn, a significant effect of replacing sucrose with inulin or polydextrose was observed in the case of the maximum viscosity, the values of which decreased with increasing amount of inulin, while in the case of polydextrose these changes were observed as a fluctuating tendency (Table 2). These observations confirm the results obtained by Witczak et al. [9], who found that the addition of inulin caused a significant decrease in starch viscosity during pasting. Moreover, the authors observed declining starch paste viscosity with increasing concentration of inulin. Additionally, Wang et al. [10] observed a decrease in the viscosity of inulin-containing starch paste, with inulin of medium degree of polymerization having the highest effect. Diversified influence of the presence of prebiotic polysaccharides on pasting characteristics of potato starch was also found in the case of the HPV, however, only the influence of the type of polysaccharide was found to be significant. The presence of inulin did not significantly affect this viscosity, while the usage of polydextrose into the system caused an increase in the HPV, regardless its concentration.

The increase in the HPV value in systems containing polydextrose could be explained by the structure of this polymer, i.e., a significant degree of branching, which even at elevated temperature could disturb the sample flow, resulting in recorded high viscosity. A significant effect of prebiotic polysaccharides was also observed in the case of viscosity breakdown (BD) during heating (Table 2). The presence of inulin in the systems significantly lowered the BD values, which resulted from its significant effect on the reduction of the peak viscosity (PV) with a negligible effect on the hot paste viscosity (HPV). In the case of presence of polydextrose in the systems, the trend of changes in the BD value was analogous to that for the peak viscosity (PV), i.e., the highest BD was found for the PD67 sample of the highest PV. The replacement of a portion of sucrose content by inulin had no significant effect on the final viscosity (FV) of the desserts (see Table 2). On the other hand, the desserts containing polydextrose were characterized by higher final viscosity values in comparison to the control. However, no tendency in changes of the FV related to the concentration of this polysaccharide was found. The presence of prebiotic polysaccharides in the desserts had a significant effect on the setback in viscosity (SB) only in the case of inulin. When sucrose was replaced by two-thirds of inulin or it was completely replaced, a significant decrease in the SB values were observed, which resulted from decreases in the final viscosity with insignificant changes in the HPV (Table 2). The results of two-way analysis of variance showed that both the level of sucrose substituting agent and its type as well as the interaction of these two factors had a significant impact on the PV, BD and SB values (Table 2). In the case of the HPV and FV parameters, the interaction of the level of sucrose substituting agent with type of the latter did not significantly affect the obtained results. On the other hand, none of the factors and their interaction had an impact on the values of the pasting temperature (PT) (Table 2).

### 3.2. Flow Behaviour

**Figure 2:**
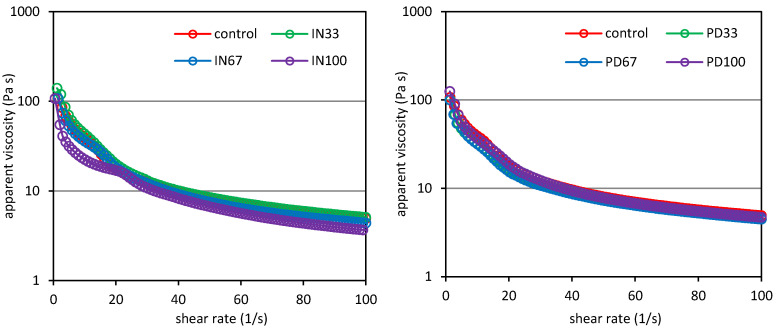
Viscosity curves of potato starch based desserts: without steviol glycosides (control), with inulin (IN33, IN67, IN100), with polydextrose (PD33, PD67, PD100).

Figure 2 shows the viscosity curves of the analyzed starch-milk desserts containing inulin or polydextrose as sucrose replacers, and parameters of the power law model describing the experimental curves are summarized in Table 3. The results show that there were differences between the samples in terms of flow properties. All the desserts tested showed a non-Newtonian shear thinning behaviour, which means that their apparent viscosity decreased with increasing shear rate. Such rheological character of multicomponent starch-containing systems is consistent with previous literature data [10,13,24]. The phenomenon of shear thinning of starch paste is caused by the breakdown of the bonds between the biopolymer molecules and orientation of the polysaccharides in the direction of flow. In consequence, this leads to a reduction in the resistance of a starch system to the applied shear stress and manifests by decreasing of apparent viscosity with an increase in a shear rate [25]. The values of the power law model parameters presented in Table 3 show the diverse influence of the presence of prebiotic polysaccharides in the starch-milk desserts on their flow characteristics.

Replacing sucrose with inulin in the amount of one third of its mass resulted in a significant increase in the value of the consistency coefficient (*K*), which reflects the initial viscosity of the material. Additionally, a simultaneous significant decrease in the value of the flow behaviour index (*n*) indicating an increase in a pseudoplasticity of the dessert was determined. In turn, the complete replacement of sucrose and the introduction of inulin instead resulted in a substantial decrease in the *K* value and a significant increase in the *n* value (Table 3). Substitution of part or a total sucrose with polydextrose resulted in slight changes in the values of both the consistency coefficient and the flow behaviour index. The results of two-way analysis of variance showed that the type of sucrose substituting agent had no significant impact on the values of the rheological parameters determined, while the level of substitution had a significant impact. 

Various effects of the prebiotic polysaccharides on the flow properties of the starch pastes are obviously due to different structure and molecular weight of the starches. Aidoo et al. [16] investigated the combinations of inulin and polydextrose and found that the increasing proportion of inulin causes an increase in plastic viscosity and a decrease in value of a yield stress of chocolate. In turn, Gao et al. [26] studied the possibility of replacing sucrose with stevia in dough formulation and found no significant effect of such operation on viscosity of the dough and starch pasting, while they reported that the substitution of sucrose with inulin resulted in a significant increase in viscosity of the dough. In the case of inulin, the degree of polymerization is of key importance for the rheological characteristics of the system. Kou et al. [27] investigated the influence of inulin preparations with different degrees of polymerization on the rheological properties of starch pastes. The authors found that a short-chain inulin causes the resulting starch paste to be characterized by lower consistency coefficient value and greater flow index value, while using of a long-chain inulin results in increased consistency coefficient value, and thus increased apparent viscosity, with no effect on the flow index value. In our study, the outcomes of two-way analysis of variance showed that the type of sucrose substituting agent had no significant impact on the values of both the consistency coefficient and the flow behaviour index (Table 3).

### 3.3. Viscoelastic Properties

Starch paste of appropriate concentration, as a result of cooling, creates a densely packed gel structure, in which water molecules and the substances dissolved in it are being enclosed. In more complex systems, such as starch-based desserts, in addition to starch, other ingredients or recipe additives, e.g., milk proteins and sucrose, contribute to the formation of the gel structure. Analysis of viscoelastic properties allows one the assessment of the influence of individual components, additives or external conditions on the rheological characteristics of multi-component food systems.

**Figure 3:**
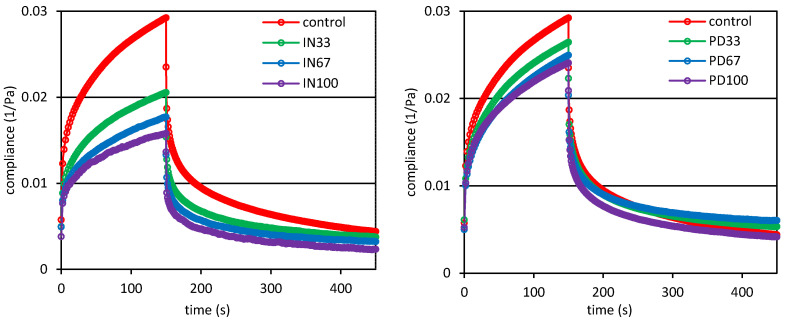
Creep and recovery curves of potato starch based desserts: without steviol glycosides (control), with inulin (IN33, IN67, IN100), with polydextrose (PD33, PD67, PD100).

Analysis of the viscoelastic properties of starch-based desserts can rely on the assessment of the results of a static creep and recovery test. The creep and recovery curves for the control sample and the desserts with inulin or polydextrose are shown in Figure 3. A typical creep curve shows the change in compliance with time at constant stress, while the recovery phase, i.e., the phase after the applied stress is removed, shows the amount of released energy, which was stored in the material structure. The highest values of creep compliance were found for the control dessert, while the presence of inulin or polydextrose resulted in the reduced susceptibility of the samples to the applied stress, which indicates strengthening of the dessert structure. The experimental curves (Figure 3) also indicate that the higher inulin concentration in the system was, the more strengthened was the structure of the material, and finally the system was less susceptible to the stress. The decrease in creep compliance also occurred in the case of replacing sucrose with polydextrose; however, it was at a lower extent compared to the samples containing inulin.

These observations are confirmed by the parameters of the Burgers model, which was used to describe the experimental curves (Table 4). In the case of the desserts with inulin, a significant decrease in the values of the instantaneous and viscoelastic compliances was observed compared to the control sample with a simultaneous significant increase in the values of zero shear viscosity.

The retardation time indicates the time at which the strain increases to a value of 63% of its total value. The statistical differentiation of the retardation time within the compared samples was low; however, significant increase in value of this parameter was observed for the dessert in which a total sucrose was replaced with inulin (IN100). Substitution of sucrose with polydextrose at all levels resulted in a decrease in the value of viscoelastic compliance as compared to the control sample, and a significant decrease in the value of instantaneous compliance was found only in the case of the highest ratio of polydextrose in the dessert (PD100). Presence of polydextrose in the systems had little effect on the value of zero shear viscosity and only for the PD67 sample a significantly higher value as compared to the control one was found. The starch-milk desserts with polydextrose were characterized by insignificantly lower values of retardation times as compared to the control sample. The results of two-way analysis of variance showed that both the level and the type of sucrose substituting agent had a significant impact on the compliance values (Table 4). On the other hand, the zero-shear viscosity and retardation time were significantly influenced by both the level and type of sugar substituting agent and the interaction of these two factors (Table 4).

**Figure 4:**
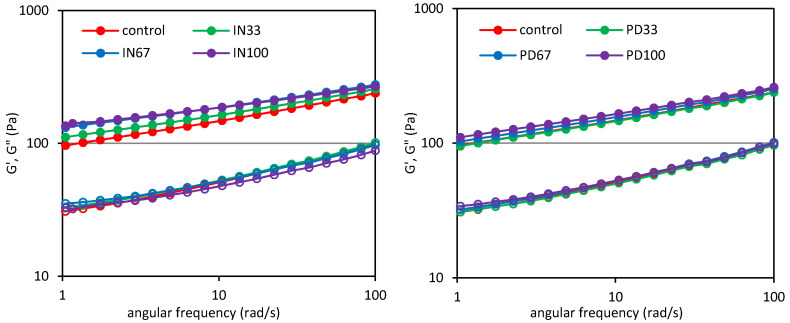
Sweep frequency curves of potato starch based desserts: without steviol glycosides (control), with inulin (IN33, IN67, IN100), with polydextrose (PD33, PD67, PD100); G′-filled markers, G″-empty markers.

**Figure 5:**
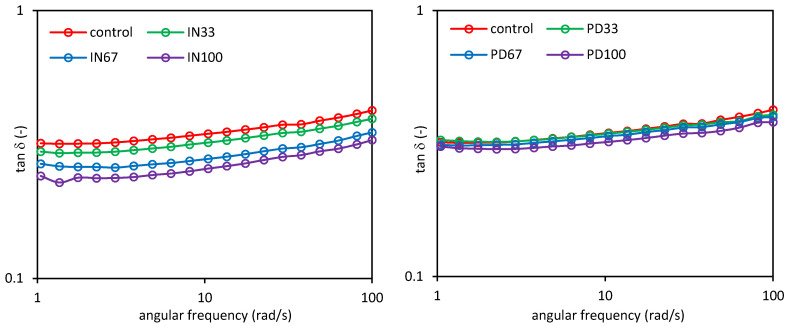
Tangent of the phase shift angle of potato starch based desserts: without steviol glycosides (control), with inulin (IN33, IN67, IN100), with polydextrose (PD33, PD67, PD100)

Figure 4 shows the sweep frequency curves as the dependence of the storage (*G*′) and loss (*G*″) moduli on the angular frequency (*ω*) for the control dessert and these with inulin (IN) or polydextrose (PD). In all cases, the values of the storage modulus were higher than those of the loss modulus (*G*′ > *G*″), indicating the predominance of elastic properties over the viscous ones, wherein a significant dependence of the both moduli on the angular frequency was found. These observations are confirmed by the values of the tangent of phase shift angle (*tan δ*), which ranged from 0.1 to 1.0, as shown in Figure 5.

This means that the analyzed desserts were characterized by typical weak gel properties, which are characteristic for many food systems. Substitution of sucrose with inulin resulted in a significant increase in the *G*′ value with the proportion of inulin in the system. However, the influence of inulin on the *G*″ value was small. This is confirmed by the value of the tangent of phase shift angle, which decreased significantly with the amount of inulin in the dessert (Figure 5). It indicates strengthening its structure and an increase in the proportion of elastic properties. In turn, the use of polydextrose had a substantially lower impact on the viscoelastic properties of the desserts, because only the concentration of this polysaccharide caused a significant increase in the *G*′ value and a decrease in the *tan δ* value. These observations are confirmed by the values of parameters of the power law models describing the experimental curves (Table 5). Substitution of sucrose with inulin resulted in a significant increase in the value of the *K*′ parameter representing the initial *G*′ value, with a simultaneous decrease in the value of the *n*′ parameter. This indicates higher flexibility of the material and a lower dependence of the *G*′ modulus on the angular frequency. On the other hand, the use of polydextrose had a significant effect on the increase in the value of the *K*′ parameter, and thus, the increase in the *G*′ value, only at the highest concentration (PD100). This increase was much lower than in the case of the use of inulin. The values of the *n*′ parameter of the desserts containing polydextrose decreased with increasing concentration of the latter, indicating a lower dependence of the *G*′ modulus on the angular frequency. However, the decrease was much lower than that of the desserts containing inulin. In the case of the *K*″ parameter, which reflects the initial value of the loss modulus, its slight increase was found compared to the control sample. The statistical differences in the values of this parameter within the samples were, in most cases, insignificant.

On the other hand, the values of the *n*″ parameter were lower compared to the control sample. Nevertheless, the decrease in the value of this parameter did not increase with the concentration of the prebiotic polysaccharide in the dessert. The results of two-way analysis of variance showed that both the level and the type of sucrose substituting agent had a significant impact on the values of parameters of the power law models describing the mechanical spectra, with exception of the *K*″ parameter (Table 5). Although the two methods used to analyze the viscoelastic properties of desserts have different theoretical principles, their results often correlate with each other. A linear negative correlation was found between the instantaneous compliance (*J*_0_) and the *K*′ (r = –0.97) and *K*″ (r = –0.69) parameters, while the *n*′ and *n*″ parameters positively correlated with the instantaneous compliance (r = 0.94 and r = 0.95, respectively). Similarly, the values of viscoelastic compliance (*J*_1_) negatively correlated with the *K*′ and *K*″ parameters (r = –0.96 and r = –0.61, respectively), while positively correlated with the *n*′ (r = 0.95) and *n*″ (r = 0.94) parameters. It was also found, that the zero-shear viscosity (*η*_0_) positively correlated with the *K*′ parameter (r = 0.88) and negatively correlated with the *n*′ and *n*″ parameters (r = –0.89 and r = –0.77, respectively).

One of the key factors influencing the viscoelastic characteristics of starch foods enriched with inulin is the degree of polymerization of the latter, and thus the length of the fructan chains. Witczak et al. [9] observed that the viscoelastic properties of starch-inulin gels strongly depended on degree of polymerization of inulin. In turn, Gonzalez-Tomas et al. [18] found that the effect of different types of inulin with different chain lengths on the viscoelastic characteristics of tapioca starch-milk desserts depended not only on the concentration of inulin, but also on the presence of other dessert ingredients. The highest effect on the viscoelastic properties was observed in the case of the addition of long-chain inulin at the highest concentration, where increase in the *G′* and *G*″ values and a significant reduction in the *tan δ* value were observed. Long non-branched chains of inulin can interact with other ingredients of desserts, for example with linear amylose, and through the network of hydrogen bonds can stabilize the structure of the product and enhance the elasticity of the system. Another mechanism of interactions will take place in the case of polydextrose, the structure of which consists of randomly connected and branched short-chain polymers. It will co-create viscoelastic structures through hydrogen bonds in a limited way. Thus, the formation of structures responsible for the increase in proportion of elastic properties in the viscoelastic ones will concern less polydextrose than inulin itself. Polydextrose will generate a thickening effect resulting, among others, from a steric hindrance, a phenomenon characteristic for highly branched polysaccharides.

## 4. Conclusions

Substitution of sucrose with prebiotic polysaccharides modified the rheological characteristics of the starch-milk desserts to a degree depending on the type and level of the substituting agent. The introduction of inulin reduced the peak viscosity of starch paste, which could be related to the restriction of swelling of starch granules and to a plasticizing effect. Inulin had, however, no effect on the final viscosity of the desserts, while polydextrose caused its increase as compared to the control dessert. The desserts showed a non-Newtonian, shear-thinning flow behaviour. The use of inulin, in both the highest and the lowest concentrations, significantly changed the consistency coefficient and the flow index values, while such a phenomenon was not observed in the case of the use of polydextrose. Substitution of sucrose with inulin significantly strengthened the viscoelastic structure of the dessert, which was manifested in increased values of the storage modulus (*G′*), and, at the same time, reduced values of both the tangent of phase shift angle (*tan δ*) and susceptibility to stress in the creep phase (*J*). The effect of polydextrose on the viscoelastic characteristics of the desserts was found to be substantially lower than that of inulin itself. The outcomes obtained in the study indicate that the both prebiotic polysaccharides (inulin and polydextrose) seem to be suitable substitutes for sucrose in desserts with reduced sugar content. The influence of these compounds on the rheological characteristics of the product is governed by their structures. Inulin, being a linear polymer, can strengthen the elastic structure of a starch-milk dessert by interacting with its other ingredients, mainly through the formation of hydrogen bonds. Polydextrose, on the other hand, will exhibit a thickening effect due to its more branched structure.

## Figures and Tables

**Table 1:** Sample codes and recipe compositions per 100 g of ready desserts.

Sample Code	Starch (g)	Milk (g)	Sucrose (g)	Steviol Glycosides (g)	Inulin (g)	Polydextrose (g)
Control	7.65	84.7	7.63	0	0	0
IN33	7.65	84.7	5.07	0.009	2.56	0
IN67	7.65	84.7	2.54	0.017	5.09	0
IN100	7.65	84.7	0	0.024	7.63	0
PD33	7.65	84.7	5.07	0.009	0	2.56
PD67	7.65	84.7	2.54	0.017	0	5.09
PD100	7.65	84.7	0	0.024	0	7.63

**Table 2:** Parameters of pasting characteristics of potato starch in systems forming starch-milk desserts.

Sample Code	PT (°C)	PV (mPa·s)	HPV (mPa·s)	BD (mPa·s)	FV (mPa·s)	SB (mPa·s)
Control	73.4 ± 0.1 ^a^	3305 ± 37 ^d^	2815 ± 49 ^a^	490 ± 38 ^e^	3601 ± 72 ^a,b^	786 ± 25 ^c^
IN33	73.8 ± 0.5 ^a,b^	3248 ± 22 ^c^	2813 ± 15 ^a^	435 ± 21 ^d^	3608 ± 24 ^a,b,c^	795 ± 12 ^c^
IN67	73.5 ± 0.1 ^a^	2991 ± 21 ^b^	2843 ± 19 ^a,b^	149 ± 8 ^b^	3599 ± 23 ^a,b^	756 ± 4 ^b^
IN100	74.3 ± 0.1 ^b^	2883 ± 26 ^a^	2851 ± 23 ^a,b,c^	32 ± 7 ^a^	3571 ± 30 ^a^	721 ± 9 ^a^
PD33	74.0 ± 0.5 ^a,b^	3321 ± 25 ^d^	2901 ± 25 ^c^	420 ± 6 ^d^	3671 ± 25 ^c^	771 ± 2 ^b,c^
PD67	74.3 ± 0.1 ^b^	3506 ± 30 ^e^	2958 ± 42 ^d^	549 ± 13 ^f^	3752 ± 29 ^d^	795 ± 22 ^c^
PD100	74.1 ± 0.5 ^a,b^	3234 ± 27 ^c^	2893 ± 14 ^b,c^	341 ± 30 ^c^	3662 ± 5 ^c^	769 ± 18 ^b,c^
Two-way ANOVA—*p*						
Factor A	0.261	<0.001	0.065	<0.001	0.035	0.0020
Factor B	0.101	<0.001	<0.001	<0.001	<0.001	0.0129
Factor A × Factor B	0.054	<0.001	0.135	<0.001	0.110	0.0023

PT—pasting temperature, PV—peak viscosity, HPV—hot paste viscosity, BD—breakdown, FV—final viscosity, SB—setback; Factor A—level of substitution of sucrose with inulin or polydextrose, Factor B—type of sucrose substituting agent (inulin or polydextrose), Factor A × Factor B—interaction of Factor A and Factor B. The data represent mean value of three replications ± standard deviation. The values described with the same letters (a–f) in column are not significantly different at level of confidence of 0.05.

**Table 3:** Parameters of power law model describing flow curves of potato starch-milk desserts.

Sample Code	Consistency Coefficient, *K* (Pa∙s^n^)	Flow Behaviour Index, *n* (-)	R^2^
Control	152.7 ± 6.93 ^b,c^	0.303 ± 0.012 ^c^	0.975
IN33	221.7 ± 15.40 ^d^	0.217 ± 0.025 ^a^	0.978
IN67	157.7 ± 2.74 ^c^	0.277 ± 0.006 ^b,c^	0.973
IN100	89.0 ± 7.57 ^a^	0.370 ± 0.010 ^d^	0.969
PD33	141.5 ± 9.17 ^b^	0.287 ± 0.006 ^b,c^	0.989
PD67	157.0 ± 3.31 ^c^	0.263 ± 0.021 ^b^	0.969
PD100	166.1 ± 4.56 ^c^	0.277 ± 0.006 ^b,c^	0.984
Two-way ANOVA—*p*			
Factor A	<0.001	<0.001	
Factor B	0.745	0.088	
Factor A × Factor B	<0.001	<0.001	

Factor A—level of substitution of sucrose with inulin or polydextrose, Factor B—type of sucrose substituting agent (inulin or polydextrose), Factor A × Factor B—interaction of Factor A and Factor B. The data represent mean value of three replications ± standard deviation. The values described with the same letters (a–d) in column are not significantly different at level of confidence of 0.05.

**Table 4:** Parameters of the Burgers model describing creep and recovery curves of potato starch milk desserts.

Sample Code	Instantaneous Compliance, *J*_0_	Viscoelastic Compliance, *J*_1_	Zero Shear Viscosity, *η*_0_	Retardation Time, *λ_ret_*	R^2^
	(1/Pa)	(1/Pa)	(Pa·s)	(s)	
Control	0.01198 ± 0.00058 ^c^	0.01101 ± 0.00073 ^d^	26926 ± 1192 ^ab^	46.2 ± 5.1 ^a^	0.987
IN33	0.01025 ± 0.00143 ^b^	0.00749 ± 0.00120 ^c^	34992 ± 1308 ^c^	58.2 ± 3.2 ^b^	0.993
IN67	0.00842 ± 0.00033 ^a^	0.00581 ± 0.00047 ^b^	44058 ± 3439 ^d^	47.4 ± 1.7 ^a^	0.992
IN100	0.00874 ± 0.00036 ^a^	0.00543 ± 0.00029 ^a^	59598 ± 4213 ^e^	66.4 ± 3.4 ^c^	0.996
PD33	0.01143 ± 0.00099 ^bc^	0.00950 ± 0.00032 ^c^	25154 ± 5069 ^ab^	41.5 ± 5.2 ^a^	0.989
PD67	0.01079 ± 0.00043 ^bc^	0.00920 ± 0.00041 ^c^	31564 ± 1248 ^bc^	44.9 ± 1.9 ^a^	0.986
PD100	0.01045 ± 0.00063 ^b^	0.00857 ± 0.00017 ^c^	25514 ± 3716 ^a^	40.3 ± 5.0 ^a^	0.987
Two-way ANOVA—*p*					
Factor A	0.018	0.004	<0.001	<0.001	
Factor B	<0.001	<0.001	<0.001	<0.001	
Factor A × Factor B	0.548	0.073	0.002	0.019	

Factor A—level of substitution of sucrose with inulin or polydextrose, Factor B—type of sucrose substituting agent (inulin or polydextrose), Factor A × Factor B—interaction of Factor A and Factor B. The data represent mean value of three replications ± standard deviation. The values described with the same letters (a–e) in column are not significantly different at level of confidence of 0.05.

**Table 5:** Parameters of power law equations describing mechanical spectra of potato starch milk desserts.

Sample	*K*′	*n*′	R^2^	*K*″	*n*″	R^2^
	(Pa·s^n’^)	(-)		(Pa·s^n”^)	(-)	
Control	94.5 ± 3.1 ^a^	0.200 ± 0.000 ^e^	0.999	28.8 ± 0.98 ^a^	0.263 ± 0.006 ^d^	0.994
IN33	108.7 ± 5.3 ^b^	0.183 ± 0.006 ^cd^	0.998	30.6 ± 0.20 ^abc^	0.247 ± 0.006 ^bc^	0.990
IN67	129.7 ± 4.9 ^c^	0.160 ± 0.000 ^b^	0.999	32.1 ± 0.67 ^c^	0.223 ± 0.006 ^a^	0.985
IN100	133.7 ± 2.0 ^c^	0.147 ± 0.006 ^a^	0.998	29.9 ± 0.30 ^ab^	0.220 ± 0.000 ^a^	0.982
PD33	93.6 ± 5.6 ^a^	0.197 ± 0.012 ^d^	0.999	29.0 ± 0.84 ^a^	0.250 ± 0.000 ^c^	0.993
PD67	100.8 ± 3.4 ^a^	0.193 ± 0.006 ^e^	0.999	30.2 ± 2.14 ^abc^	0.250 ± 0.010 ^c^	0.994
PD100	108.7 ± 4.0 ^b^	0.180 ± 0.000 ^c^	0.999	31.5 ± 1.63 ^bc^	0.237 ± 0.000 ^b^	0.990
Two-Way ANOVA—*p*						
Factor A	<0.001	<0.001		0.143	<0.001	
Factor B	<0.001	<0.001		0.272	<0.001	
Factor A × Factor B	0.038	0.013		0.033	0.012	

Factor A—level of substitution of sucrose with inulin or polydextrose; Factor B—type of sucrose substituting agent (inulin or polydextrose); Factor A × Factor B—interaction of Factor A and Factor B. The data represent mean value of three replications ± standard deviation. The values described with the same letters (a–e) in column are not significantly different at level of confidence of 0.05.
